# Elastic, magnetic and electronic properties of iridium phosphide Ir_2_P

**DOI:** 10.1038/srep21787

**Published:** 2016-02-24

**Authors:** Pei Wang, Yonggang Wang, Liping Wang, Xinyu Zhang, Xiaohui Yu, Jinlong Zhu, Shanmin Wang, Jiaqian Qin, Kurt Leinenweber, Haihua Chen, Duanwei He, Yusheng Zhao

**Affiliations:** 1 Institute of Atomic and Molecular Physics, Sichuan University, Chengdu 610065, People’s Republic of China; 2 High Pressure Science and Engineering Center, University of Nevada, Las Vegas, Las Vegas, Nevada 89154, United States; 3 State Key Laboratory of Metastable Materials Science and Technology, Yanshan University, Qinhuangdao 066004, People’s Republic of China; 4 Beijing National Laboratory for Condensed Matter Physics and Institute of Physics, Chinese Academy of Sciences, Beijing 100190, People’s Republic of China; 5 Metallurgy and Materials Science Research Institute, Chulalongkorn University, Bangkok 10330, Thailand; 6 Department of Chemistry and Biochemistry, Arizona State University, Tempe, Arizona 85287, United States; 7 Department of Fundamental Courses, Qinghai University, Xining 810016, People’s Republic of China

## Abstract

Cubic (space group: *Fm*
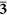
*m*) iridium phosphide, Ir_2_P, has been synthesized at high pressure and high temperature. Angle-dispersive synchrotron X-ray diffraction measurements on Ir_2_P powder using a diamond-anvil cell at room temperature and high pressures (up to 40.6 GPa) yielded a bulk modulus of *B*_*0*_ = 306(6) GPa and its pressure derivative *B*_0_′ = 6.4(5). Such a high bulk modulus attributed to the short and strongly covalent Ir-P bonds as revealed by *first – principles* calculations and three-dimensionally distributed [IrP_4_] tetrahedron network. Indentation testing on a well–sintered polycrystalline sample yielded the hardness of 11.8(4) GPa. Relatively low shear modulus of ~64 GPa from theoretical calculations suggests a complicated overall bonding in Ir_2_P with metallic, ionic, and covalent characteristics. In addition, a spin glass behavior is indicated by magnetic susceptibility measurements.

Understanding the physical properties of hard materials continues to be a motivating and active area of research^1^,^2^. Compounds between transition metals and low-Z elements (IIIA-VIA) have attracted considerable interests during the last two decades. Recently transition-metal borides (WB_4_, ReB_2_, CrB_4_, CrB, IrB_1.35_), nitrides (IrN_2_, OsN_2_, W_x_N_y_, CrN), and carbides (Re_2_C, PtC) have been investigated for their high bulk modulus and hardness^3^,^4^,^5^,^6^,^7^,^8^,^9^,^10^,^11^,^12^,^13^,^14^,^15^,^16^,^17^,^18^,^19^,^20^,^21^,^22^,^23^. However, transition-metal phosphides have received minimum attention up to date. The bonding scheme in this group can be analogous to that of their boride counterparts^24^,^25^. While the latter is characterized by sharing metal-metalloid (M-P) bonds with a strong covalent component, transition metal phosphides have strong and highly metalloid-metalloid (P-P) bonds. When compared with the bonds observed in metal nitrides and carbides, these P-P bonds are even stronger^25^. Moreover, a large number of transition-metal phosphides have been reported with varying compositions and crystal structures and rich physical properties such as catalytic functions, high hardness, thermoelectric effect, magnetism, and luminescence^25^,^26^,^27^,^28^,^29^,^30^,^31^,^32^.

A few studies have been conducted on iridium phosphide (Ir_2_P) for its synthesis routes and crystal structure. The compound was first reported in 1935^33^,^34^. Zumbusch *et al.* assigned the anti-fluorite structure for Ir_2_P^35^. Rundqvist *et al.* established the compositional systematics of platinum-metal phosphides and further examined the crystal structure of Ir_2_P with single-crystal diffraction method^33^. Raub *et al.* reported Ir_2_P exhibited a metallic behavior^36^. Sweeney *et al.* explored the feasibility of reductive hydrogen annealing of metal phosphates as a synthesis pathway to phosphides^32^. However, there are no literature data on the elastic and deformation properties of Ir_2_P because it is difficult to synthesize Ir_2_P at ambient pressure. Systematic studies of elastic properties and hardness of Ir_2_P are important to understanding the platinum phosphides as a group and finding potential pathway for their practical applications. In this work, Ir_2_P was synthesized at high pressure (P) and high temperature (T) and subsequently investigated for its hardness and elastic, electronic, and magnetic properties via measurements of *in-situ* high – P synchrotron x-ray diffraction, micro-hardness indentation, and low – T magnetic susceptibility. *First – principles* calculations were also carried out to explore the relationship between electronic and elastic properties of the titled material.

## Results and Discussion

**Figure 1:**
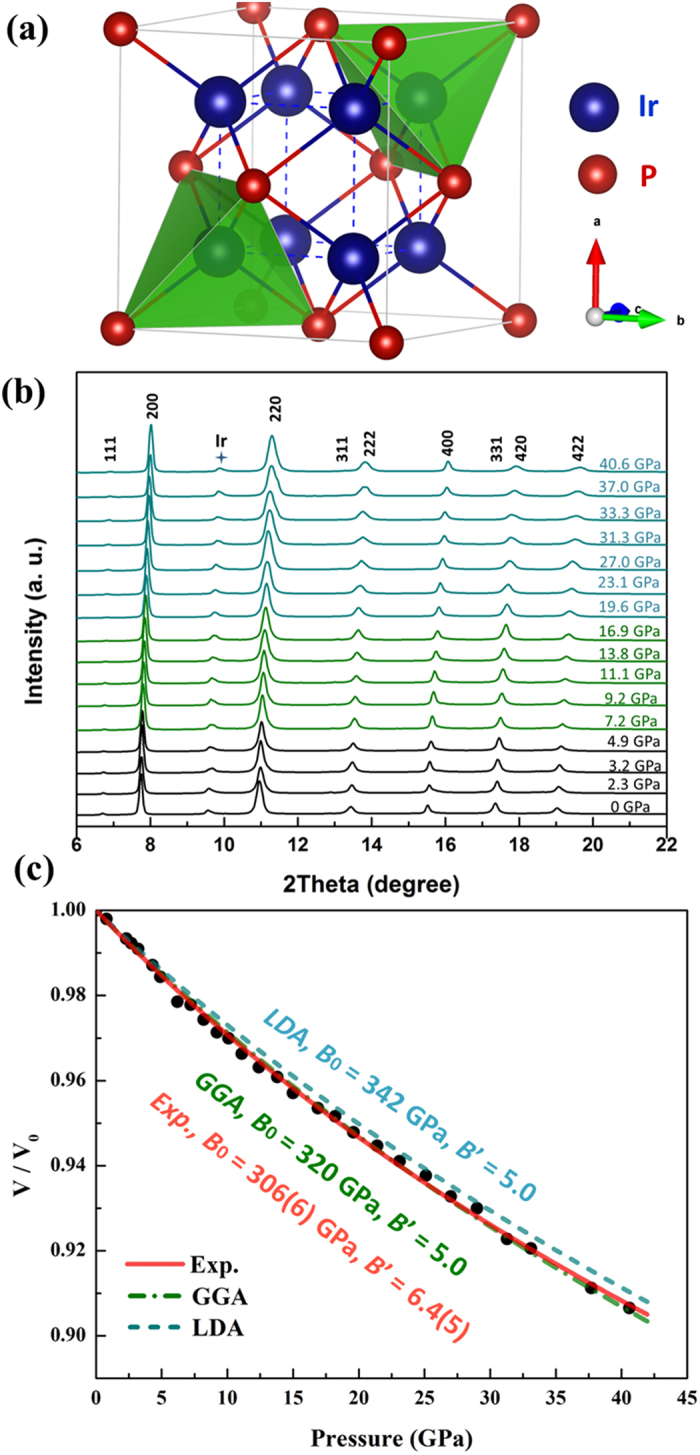
(**a**) The crystal structure of cubic Ir_2_P; (**b**) at room temperature, representative high-pressure x-ray diffraction patterns of Ir_2_P synthesized at 15 GPa/1800 °C; (**c**) the volume-pressure data fitted to the 3^rd^ order BM-EoS from experiment and calculation. Filled circles represent the experimental data points; The solid line is the EoS fit to experimental data; The dash and dash-dot lines represent the results from LDA and GGA, respectively. Error bars for all experimental data points are smaller than the size of the symbols.

At ambient conditions, Ir_2_P adopts a CaF_2_-type structure with a space group of *Fm*
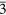
*m*^32^,^33^,^34^,^35^. As shown in Fig. 1a, each iridium atom is surrounded by four phosphor atoms and [IrP_4_] tetrahedrons are edge – sharing and form a 3D network. The refined cation-anion bond length is 2.40 Å and the cation–cation bond length is 2.77 Å.

To study the phase stability and compressibility of cubic Ir_2_P, synchrotron powder x-ray diffraction experiment was performed in a symmetric diamond anvil cell (DAC) up to 40.6 GPa at room temperature. Figure 1b shows the representative diffraction patterns as a function of pressure. A small amount of metal Ir impurity was detected in the diffraction patterns. No pressure-induced phase transition was observed, suggesting the cubic Ir_2_P is stable in the pressure range of investigation under experimental conditions. By fitting the compression data to a second and third-order Birch-Murnaghan equations of state (BM-EoS), we obtained bulk modulus *B*_0_ = 334(2) GPa with *B*_0_′ = 4 (fixed), and *B*_0_ = 306(6) GPa and *B*_0_′ = 6.4(5), respectively. Bulk modulus values from *first – principles* calculations (see Methods), *B*_0_ = 320 GPa with *B*_0_′ = 5.0 from generalized gradient approximation (GGA) and *B*_0_ = 342 GPa with *B*_0_′ = 5.0 from local density approximation (LDA) (Table 1), are consistent with the experimental results. Iridium is the second least compressible noble metal (next only to osmium)^37^. The short Ir-P bonds and presence of Ir atoms having a high density of valence electrons play key roles in limiting the lattice compression (i.e., high bulk modulus) since it is extremely difficult to shorten the distances among these atoms due to the rapidly increasing repulsive forces^9^.

**Figure 2:**
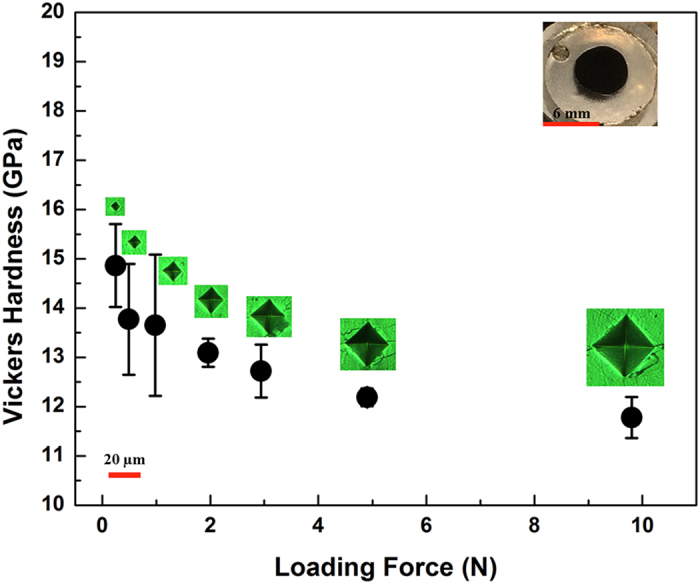
Vickers hardness of Ir_2_P as a function of applied loads from 0.245 to 9.8 N. The polished Ir_2_P sample (Inset) synthesized at 8 GPa/1400 °C contains little impurity. The scale of 20 μm applies to all indentation images.

Vickers hardness measurements were carried out on the polished surface of chunky Ir_2_P samples. Figure 2 shows the dependence of hardness on loading force. A hardness of 11.8 (4) GPa under a loading force of 9.8 N suggests Ir_2_P is considerably harder than hardened steel and some monoborides such as OsB (10.6 GPa) and RuB (8 GPa) (Table 1)^4^ and comparable to some ceramics (e.g., ZrO_2_)^38^ and WC-Co alloys in the hard regime. The hardness of Ir_2_P is also similar to that of Re_2_P (Table 1)^21^.

**Figure 3:**
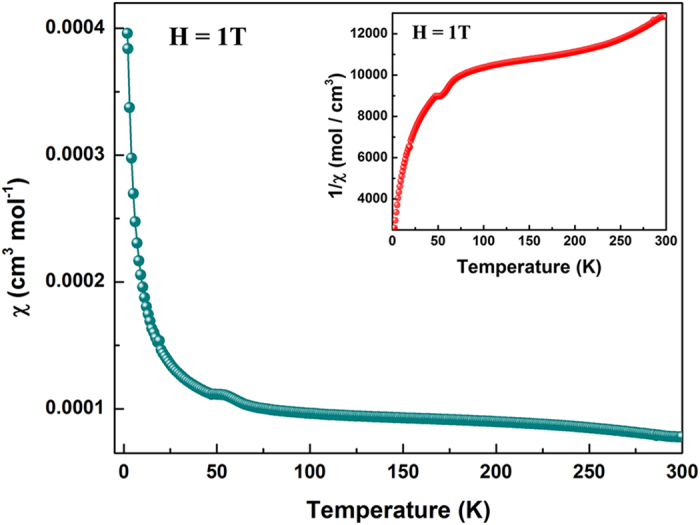
The molar susceptibility for Ir_2_P versus temperature from 2 to 300 K at 1 T (10^4^  Oe). Inset: the inverse of the molar susceptibility vs temperature.

Magnetic susceptibility measurements for Ir_2_P were performed in the temperature range of 2–300 K under a magnetic field of 1 T. Figure 3 shows the temperature dependent susceptibility. The kink at ~50 K in susceptibility revealed the possible transition of magnetic state. Moreover, the violation of Curie-Weiss law and the negative Curie-Weiss temperature (inset of Fig. 3) indicated that the spin glass behavior existed in an antiferromagnetic interaction background.

**Figure 4:**
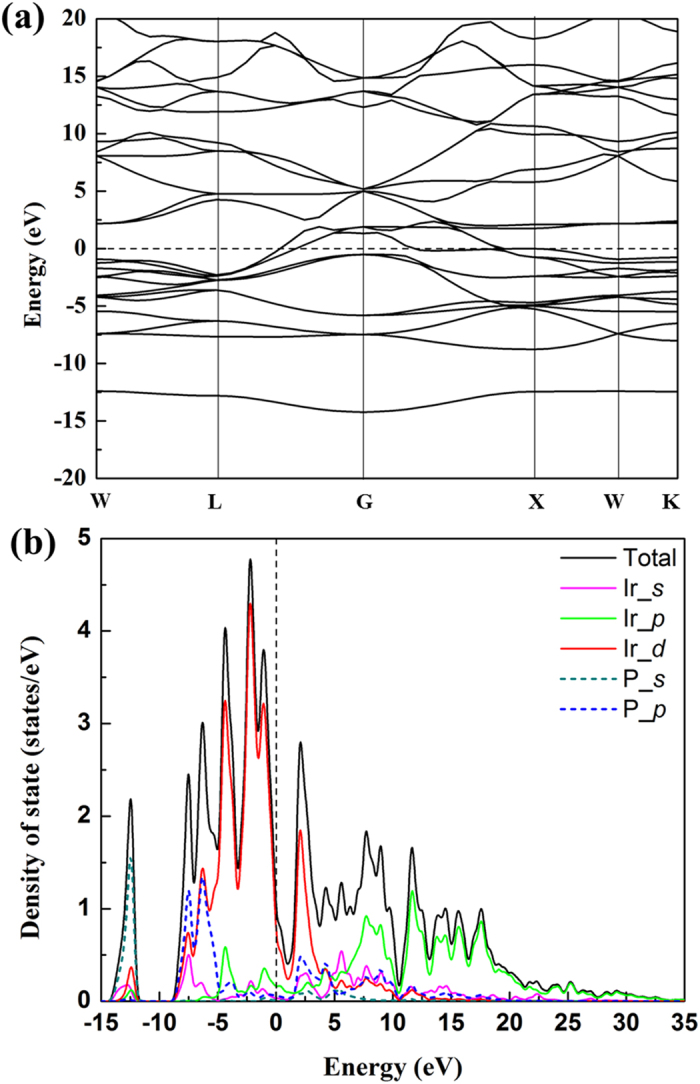
(**a**) Band structure of Ir_2_P; (**b**) Partial density of states of Ir_2_P; the pink, green and red solid curves are from Ir *s*, *p*, and *d* orbitals, respectively, and the light green and blue dashed curves are P *s* and *p* orbitals, respectively. The vertical dashed line is the Fermi level.

To correlate the chemical bonding and mechanical properties, we have performed *first – principles* calculations based on the density functional theory (DFT) using the CASTEP code with a PBE and CA-PZ exchange-correlation functional form of the GGA and LDA, respectively^39^. As shown in the calculated band structure at ambient conditions (Fig. 4a), the valence band maxima cross Fermi level and meet with the conduction band maxima between the G and X points. The overlap of valence and conduction bands indicates a metallic state for the band structure of Ir_2_P, consistent with the previous results^31^,^32^,^36^. In order to further understand the properties of Ir_2_P, the total and partial electronic densities of state (DOS) were also calculated and are shown in Fig. 4b. The Ir 5*d* and P 3*p* states of Ir_2_P dominate the DOS at the Fermi level, and the P 3*s* electrons basically lie at the bottom of valence band. The P 3*p* and Ir 5*d* orbitals having major contributions to total DOS reveal a strong hybridization between Ir *d* and P *p* orbitals. The finite DOS at the Fermi level indicates metallic behavior of Ir_2_P, consistent with the calculated band structure.

**Figure 5:**
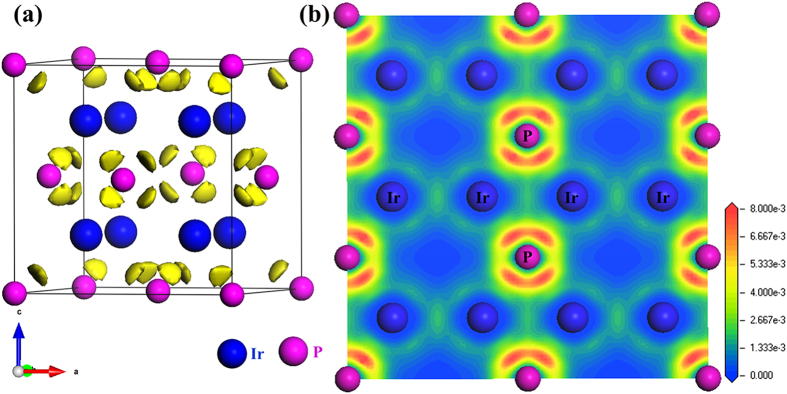
(**a**) Isosurface of electronic localization functions (ELF) for the corresponding structure with the value of 0.007 electrons/Å^3^. The large blue and small pink spheres represent Ir and P atoms, respectively. The yellow color bounded regions indicate the formation of covalent bonding networks due to charge accumulation; (**b**) ELF of (1
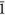
0) lattice plane.

The electronic localization function (ELF) based on the Hartree-Fock pair probability of parallel spin electron was calculated to visualize different types of bonding in solids^40^. As shown in Fig. 5a, the polar covalent bonding interactions between Ir and P are evident as the ELF maxima are strongly biased towards the P atoms. The yellow-colored electron configuration indicates a substantial accumulation of electronic charge density within the voids of crystal structure. Figure 5b clearly displays metalloid covalent bonding feature. The relatively high ELF values along Ir-P bonds mirror its covalent feature while the low ELFs between Ir ions correspond to the metallic bonding. The polar metal-metalloid (M-P) covalent bonds of short distance (2.40 Å) should result in relatively incompressible tetrahedra, which form a 3D-network through edge-sharing that further enhances Ir_2_P’s ability to resist compression. Moreover, the interlaced Ir-Ir metallic bonds as in metal Ir are difficult to be shortened under pressure. All these factors play a positive role for the high incompressibility of Ir_2_P. On the other hand, the weak Ir-Ir metallic bonds make the structure susceptible to shear deformation under stress, resulting in the relatively low shear modulus (Table 1) and hardness of Ir_2_P shown in Fig. 2.

## Conclusions

In summary, we synthesized cubic Ir_2_P at high pressure and high temperature (HPHT). In the pressure range of 0 to 40.6 GPa, Ir_2_P has a high bulk modulus of *B*_*0*_ = 306(6) GPa with *B*_0_′ = 6.4(5). It has a Vickers hardness of 11.8(4) GPa under a loading force of 9.8 N. These results are in consistence with the *first – principles* calculations that suggest the strong polar covalent bonding between Ir and P atoms leads to the incompressibility of Ir_2_P. The metallic Ir bilayers are presumably responsible for the weakest paths under shear deformation. The temperature-dependent molar susceptibility indicates the spin glass behavior in an antiferromagnetic interaction background in Ir_2_P.

## Methods summary

### HPHT synthesis

Ir_2_P was synthesized using a mixture of Ir powder (purity 99.9%) and red phosphorus powder (purity 99.999%) with a molar ratio of Ir : P = 2 : 1 under high pressure/temperature conditions. The syntheses were carried out in a two-stage multi-anvil apparatus based on a DS 6 × 8 MN cubic press^41^,^42^ and a Walker-type multianvil press at Arizona State University^43^. The 14/8 sample assembly, consisting of a 14 mm (edge length) MgO octahedron, a ZrO_2_ thermal insulator and a Ta heater, was compressed by eight cubic WC anvils, each with 8-mm corner truncation (edge-length). Pressures were estimated based on the calibration established by phase transitions in ZnTe, ZnS, and GaAs at room temperature, and temperatures were measured *in-situ* with a Pt6%Rh–Pt30%Rh or Re5%W-Re25%W thermocouple. The samples were first compressed to targeted load, and then heated with a rate of about 100 °C /min to desired temperature and hold for 30 min. The pressure was released after the temperature was quenched to room temperature. The recovered cylindrical samples have a diameter of ~3 mm and a height of ~3 mm.

### Characterization methods

The recovered samples were characterized by x-ray diffraction with Cu Kα radiation source. High-pressure *in*-*situ* powder x-ray diffraction experiments were performed using a symmetric diamond anvil cell (DAC) with a culet size of 300 μm at 16-IDB of the High Pressure Collaborative Access Team (HPCAT), Advanced Photon Source (APS), Argonne National Laboratory (ANL). The Ir_2_P powders were loaded into a pre-indented gasket (steel) hole in diameter 170 μm. A few ruby balls were also loaded in the sample chamber to serve as the internal pressure standard^44^. Neon was used as pressure-transmitting medium to improve hydrostatic pressure conditions for the sample. The incident x-ray beam of wavelength 0.3738 Å was focused to approximately 5 μm × 7 μm. The distance between image plate and sample is 182 mm. The 2D patterns of intensity versus 2*θ* were obtained by using the FIT2D software^45^. Vickers hardness was measured on a well – sintered polycrystalline sample under different loads of 25, 50, 100, 200, 300, 500, and 1000 g by using a Micromet–2103 hardness tester (Buehler, USA). Under each applied load, the measurement was performed with a dwelling time of 15 s, and was repeated 5–10 times to obtain statistically improved averages. The low temperature magnetic susceptibility measurements were performed in a Quantum Design superconducting quantum interference device (SQUID) an external field of 1 T.

### Computation details

*First – principles* calculations based on density functional theory (DFT) were performed in the CASTEP code with a PBE and CA-PZ exchange-correlation functional form of the GGA and LDA, respectively^39^. The plane-wave cut-off energy was 500 eV, and the Brillouin-zone sampling was performed with the Monkhorst-Pack grid with a k-points sampling of 7 × 7 × 7. The 5*d*^7^6*s*^2^ and 3*s*^2^3*p*^3^ were taken as valence electron for Ir and P atoms, respectively. Broyden–Fletcher–Goldfarb–Shanno (BFGS) scheme was considered as the minimization algorithm^46^. The bulk modulus, shear modulus, Young’s modulus, and Poisson’s ratio were estimated by using the Voigt-Reuss-Hill approximation^47^.

## Additional Information

**How to cite this article**: Wang, P. *et al.* Elastic, magnetic and electronic properties of iridium phosphide Ir2P. *Sci. Rep.* **6**, 21787; doi: 10.1038/srep21787 (2016).

## Figures and Tables

**Table 1:** Elastic properties and hardness of Ir_2_P and analogous hard materials.

Compounds	Method	*B*_*0*_(GPa)	*B*′_0_	*G*(GPa)	*E*(GPa)	*ν*	*B*/*G*	*Hv*_Exp_ (GPa)	*Hv*_Theor_ (GPa)	References
Ir_2_P	Exp.	306(6)	6.4(5)	—	—	—	—	12		This work
	Exp.	334	fixed 4.0	—	—	—	—			This work
	GGA	320	5.0	42	661	0.154	7.6			This work
	GGA	334	fixed 4.0	—	—	—	—			This work
	LDA	342	5.0	64	693	0.162	5.3			This work
	LDA	355	fixed 4.0	—	—	—	—			This work
Re_2_P	Exp.	304 (1)	6.7(1)	—	—	—	—	13		ref. 24
	GGA	322.9(2)	4.5(0)	—	—	—	—			ref. 24
OsB	Exp.	431(23)	5.3(2)	—	—	—	—	10.6		ref. 4
	GGA	360	4.4	231	572	0.236	1.6		16.2	refs 12, 18
RuB	Exp.	261(28)	5.2(3)	—	—	—	—	8		ref. 4
RuB_1.1_	GGA	307	—	186	464	0.248	1.7	10.6	31.2	ref. 19
CrN	Exp.	257(5)	fixed 4.0	—	—	—	—	13		ref. 21
	LDA+U	255	—	—	—	—	—		1.2	refs 13, 22

Elastic Constants, the Shear Modulus *G*, Young Modulus *E,* Poisson’s ratio ν, theoretical calculation hardness *Hv*_Theor_ and experimental Vickers hardness *Hv*_Exp_.
